# What Does Text Mining of Reddit Forums Reveal About Factors Surrounding Mental Health in Singapore?

**DOI:** 10.2196/72959

**Published:** 2025-10-31

**Authors:** Charles Alba, Abel Beng Heng Ang, Vahid Abbasian, Gerard Chung

**Affiliations:** 1 Division of Computational and Data Sciences, Washington University in St. Louis, 1 Brookings Drive, St Louis, MO, 63130, United States, 1 (314) 935-5000; 2 Institute of Data Science, National University of Singapore, Singapore, Singapore; 3 Imaging Science Program, Washington University, St Louis, MO, United States; 4 Department of Social Work, Faculty of Arts and Social Sciences, National University of Singapore, Singapore, Singapore

**Keywords:** mental health, online forums, text mining, Singapore, Reddit, infodemiology

## Abstract

Text mining of mental health-related posts from Singapore-based Reddit communities uncovered 10 themes, with loneliness, access to affordable mental health care, and education challenges emerging as the most prevalent, alongside rising discussions on social isolation and emotional struggles, as well as access to mental health support and services.

## Introduction

A 2023 national survey reported a sharp rise in poor mental health among young Singaporean adults compared to 2017 [1], while another found that 46% of Singaporeans regarded mental health as the top health challenge [2], highlighting the need for effective interventions and policies.

In response, a recent white paper [3] recommended stronger policies, improved stakeholder collaboration, and greater public awareness. However, these stemmed from household surveys and interviews that struggle to capture real-time needs due to factors like stigma.

As online forums provide a complementary, timely, and open setting, this study aims to leverage Reddit forums to identify key factors and trends that shape Singapore’s mental health discourse, with the goal of helping practitioners and policymakers refine strategies and disseminate targeted recommendations.

## Methods

### Ethical Considerations

Our study was not subject to the Internal Review Board (IRB) federal definitions, as confirmed by Washington University’s IRB board (#202509029). Our study is not considered to meet federal definitions under the jurisdiction of an IRB and falls outside the purview of the Human Research Protect Office as the data was obtained from social media posts consistent with the Reddit terms of use applicable at the time. All the secondary data used were anonymous.

### Data

Reddit posts spanning January 2015 to December 2022 were retrieved from four Singapore-based subreddits – r/askSingapore, r/singapore, r/singaporeR, and r/singaporeRaw.

### Methods Used

Chain-of-thought prompting [4] first identified posts relevant to mental health. Dynamic BERTopic [5] uncovered topics and tracked their evolution over time, allowing us to observe topical shifts in discussions of mental health across different years. BERTopic embeds each post using language models, clusters semantically similar posts, and represents each cluster as keywords to form coherent topics. Finally, Linguistic Inquiry and Word Count analysis [6] generated psychological insights from posts among each identified topic. Prompts and parameters are detailed in Multimedia Appendix 1.

## Results

In total, 2,783/379,787 (0.73%) posts were identified as mental health-related. BERTopic analysis uncovered 10 distinct key topics (Table 1), which can be grouped into several categories: (1) social isolation and emotional struggles, such as loneliness (Topic 0) and relationship challenges (Topic 8); (2) access and support to services, including affordability (Topic 1) and assistance in dealing with depressive symptoms (Topic 5); (3) career and education pressures (Topics 2, 3, and 6); and (4) tragedies from personal and global events, such as assaults (Topic 7) or COVID-19 (Topic 4).

**Figure 1.**
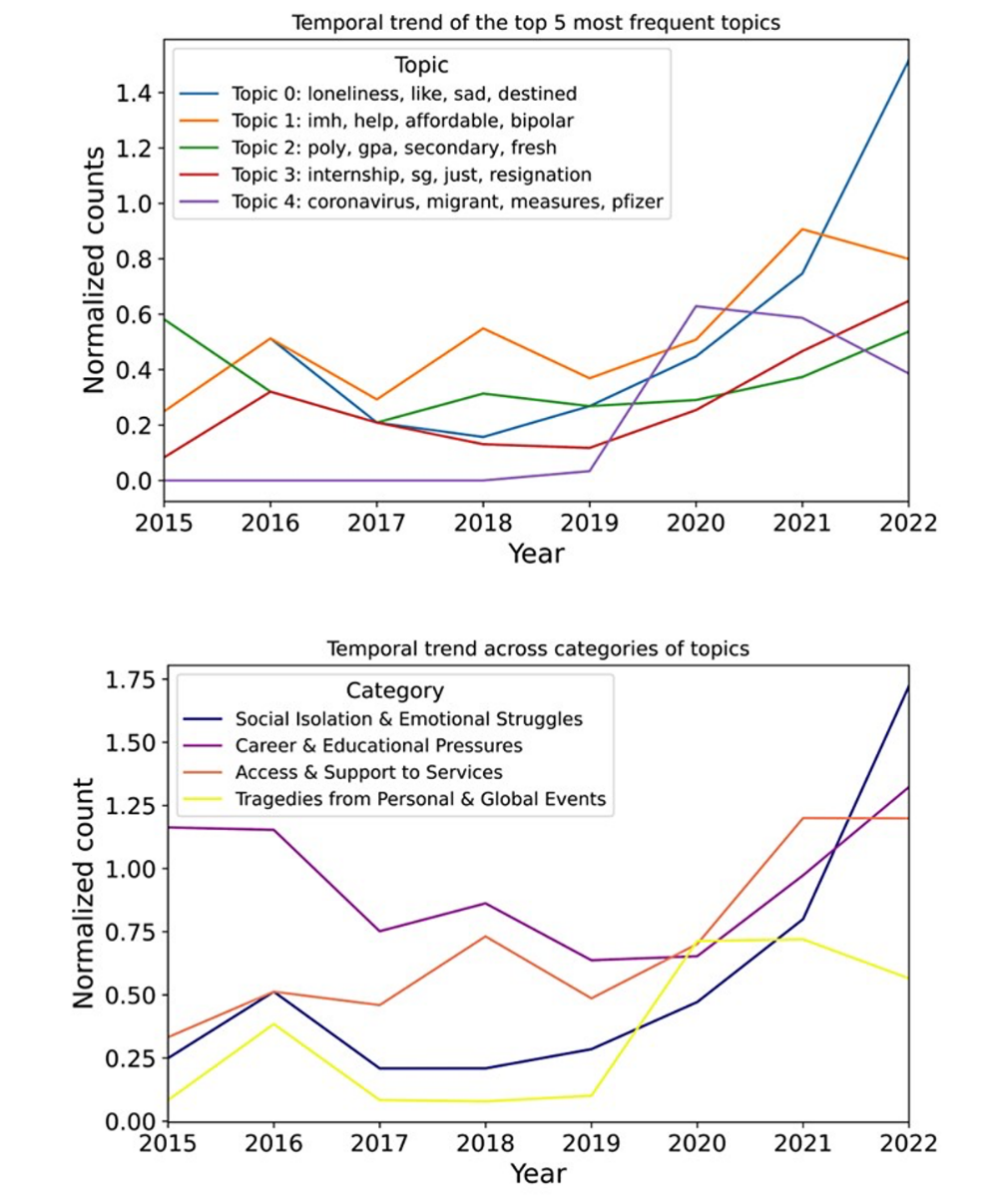
Temporal trends of the most frequent topics (top) and topic categories (bottom) identified through BERTopic analysis of Singapore-based Reddit discussions on mental health from 2015 to 2022.

Trends-wise, Figure 1 suggests that loneliness (Topic 0) has exponentially risen since 2019, while assistance with affordable care (Topic 1) has steadily increased in the same period. The counts are normalized as$\frac{{\left(\#\;\text{documents in topics}\right)}_{t}}{{\left(\#\;\text{documents across all sub-reddits}\right)}_{t}}$ where *t* represents the year, to account for changes in Reddit’s popularity among Singaporeans over time. Conversely, discussions surrounding COVID-19 (Topic 4) declined after 2020, stemming from a return to normalcy. School-and career-related struggles (Topics 2 and 3) have fluctuated since 2015 but appear to rise since 2019.

**Table 1.** Topics identified through BERTopic analysis of posts classified as relevant to mental health using chain-of-thought prompting across four Singapore-based subreddits (r/askSingapore, r/singapore, r/singaporeR, and r/singaporeRaw) spanning January 2015 to December 2022.

Topic	Count	Topic keywords	Interpretation	Representative text	Categories
0	241	loneliness, like, sad, destined	Loneliness	“Is it normal to feel unlovable… was previously ok w making friends w my friends… but … feeling lonely and depressed”	Social Isolation & Emotional Struggles
1	229	imh^a^, help, affordable, bipolar	Access to affordable mental health care	“... anyone know where I can find affordable therapy in Singapore? I would prefer private therapists or NGOs, not referrals from polyclinics or IMH… I’m looking for help with anxiety …”	Access & Support to Services
2	136	poly, gpa, secondary, fresh^b^	School-related struggles	How to transfer to another secondary school … I don’t feel accepted here… My grades are mediocre… My score is miserable… I really hate my life…	Career & Education Pressures
3	126	internship, sg, just, resignation	Career-related struggles	“Anxiety and stressed about work… I get so anxious and stressed about working, whether I am able to do what I am told or also able to understand and deliver on my own… ”	Career & Education Pressures
4	126	coronavirus, migrant^c^, measures, Pfizer	COVID-19	“Who here is feel burn out and slightly depressed because of covid...”	Tragedies from Personal & Global Events
5	86	attacks, eating, ptsd, antidepressants	Symptoms of depression	“Anxiety attacks support… looking for someone to talk to about anxiety attack”	Access & Support to Services
6	82	pes, nsf, bunk, depression^d^	Struggles with military (national) service	“Downing PES status… been in the army for about six months now… been undergoing bouts of depression… been having suicidal ideations … should i tell my commanders/MO…”	Career & Education Pressures
7	36	heard, shes, assaulted, families	Family assault	[Traumatic personal experience of assault]	Tragedies from Personal & Global Events
8	24	breakup, attachment, unavailable, sensitive	Relationship challenges	“How to deal with breakup… Just broke up with my gf recently, it hurts so bad … my mechanism with dealing with such depressing problem is actually unhealthy…”	Social Isolation & Emotion Struggles
9	10	scam, valuing, transactions, ocbc^e^	Scam incidents	“Just got scammed.... but too scared to go to the police to get help... I have been in a weirdly long manic episode…”	Tragedies from Personal & Global Events
-1	1687	man, help, singaporeans	Outlier	Outlier	Outlier

a Institute of Mental Health (IMH) is the only tertiary hospital in Singapore specializing in psychiatry.

b Poly indicates Polytechnic; Secondary indicates Secondary School.

c Referring to COVID-19 outbreaks that took place in migrant dormitories.

d Physical employment standard (PES) is used to determine an individual’s military service vocation based their medical fitness and condition; full-time national serviceman (NSF) refers to individuals serving mandatory 2-year military service.

e Overseas-Chinese banking corporation (OCBC) is one of the largest local banks in Singapore.

Among categories, topics related to “Social Isolation and Emotional Struggles” and “Access and Support to Services” are on the rise, with the former increasing exponentially since 2017.

Linguistic Inquiry and Word Count analysis revealed homogeneous distributions across topics in terms of psychological states and motives, with most attributes falling at or below the average levels observed in the broader Reddit corpus (Multimedia Appendix 2). “Allure” remains an exception with a divergence observed; topics like COVID-19 (Topic 4), symptoms of depression (Topic 5), and national service (Topic 6) fall substantially below the Reddit average, while other topics display slightly elevated levels.

Among the most prevalent topics, loneliness (Topic 0) and access to affordable mental health care (Topic 1) show sustained increases. Categorization of all topics (Table 1) indicates that discussions related to social isolation and emotional struggles, as well as access and support to services, have risen consistently.

## Discussion

Our findings reveal that loneliness, access to affordable care, and school-and career-related struggles are most prominent, with the former two on the rise. Our results can complement existing efforts, like the recently launched white paper [3], providing insights for practitioners and policymakers. Rising loneliness (Topic 0) and access to affordable services (Topic 1) highlight the need for awareness campaigns on social isolation, cost-effective treatment, technology-enabled care, and stronger family and community support. At the micro-level, addressing affordability could reduce previous findings of treatment gaps [7], while targeting loneliness could help counselors tailor interventions. Similarly, Linguistic Inquiry and Word Count analysis suggests that above-average allure across most topics may reflect societal pressures related to attractiveness.

Reddit data are limited by demographic bias and difficulty capturing cultural nuance. Yet its anonymous discussions reveal needs overlooked in surveys, remaining prone to social desirability bias.

Future research encompasses integrating this Reddit analyses with surveys and other sources for comprehensive recommendations on Singapore’s mental health needs. Our study demonstrates how digital data captures lived experiences that complement demographic trends, enabling more responsive and inclusive strategies.

## Supplementary material

10.2196/72959 Multimedia Appendix 1 Chain-of-thought evaluation, promts and parameters, and BERTopic parameters.

10.2196/72959 Multimedia Appendix 2 Linguistic Inquiry and Word Count analysis results.

## Abbreviations

IRB: Internal Review Board
