# Supplementary material for: What Does Text Mining of Reddit Forums Reveal About Factors Surrounding Mental Health in Singapore?
Source: J Med Internet Res. 2025 Oct 31;27:e72959. doi: 10.2196/72959 (PMC12578359; doi:10.2196/72959)
Supplement: Multimedia Appendix 1 [file jmir-v27-e72959-s001.docx]

## Multimedia Appendix 1.

## Supplementary Methods

### Chain-of-thought evaluation

To evaluate the chain-of-thought classifications of Reddit posts, we randomly selected 323 Reddit posts for annotation. This sample size was selected based on the formula defined by Alba et al. [1], which dictates the minimum annotated sample size required to appropriately evaluate and train a text classifier. We set $\pi=0.8, Z_{\frac{\alpha}{2}}=1.96, E=0.05$. We hired 3 annotators (via the annotation platform Prolific) to independently annotate each sampled text and determine if it was relevant to mental health. Table S1 reflects the intra-class correlation (ICC) scores, reflecting a strong level of agreement among annotators (ICC(2,3)=0.79, 95% CI [0.75, 0.83], p < .001).

Using the majority vote of three annotators as the ground-truth labels, we then evaluated the language model’s classification performance. As reflected in Table S2, the model achieved exceptional performances, with a weighted F1-score of 98%.

Table S1. Intra-class correlation (ICC) scores across 3 independent annotators in labeling 323 sampled Reddit posts to determine if it was

| **Type** | **Description** | **ICC** | **F-score** | **p-value** | **95% CI** |
| --- | --- | --- | --- | --- | --- |
| ICC1k | Average raters absolute | 0.790251 | 4.767601 | 9.92*10^-64^ | [0.75 0.83] |
| ICC2k | Average random raters | 0.791048 | 4.855946 | 4.70*10^^-65^ | [0.75 0.83] |
| ICC3k | Average fixed raters | 0.794067 | 4.855946 | 4.70*10^-65^ | [0.75 0.83] |

Table S2. Classification performance of the chain-of-thought language model. Ground truth was defined by the majority vote of three annotators and compared against the model’s predictions.

| **Type** | **Precision** | **Recall** | **F1-score** |
| --- | --- | --- | --- |
| accuracy | 0.98452 | 0.98452 | 0.98452 |
| macro avg | 0.932614 | 0.908497 | 0.920164 |
| weighted avg | 0.984163 | 0.98452 | 0.984298 |

### Chain-of-thought prompts and parameters

Table S3. Details of prompts and corresponding parameters applied in chain-of-thought prompting for identifying Reddit posts relevant to depression.

| **Type** | **Details** |
| --- | --- |
| Model | meta-llama/Meta-Llama-3.1-8B-Instruct |
| Temperature | 0.1 |
| max_new_tokens | 128 |
| set_seed | 42 |
| System Prompt | ‘‘‘You are tasked with analyzing social media posts for signs of clinical depression strictly for classification purposes. You are not required to provide advice or intervention, only to analyze and classify based on the given criteria.’’’ |
| Message Prompt | ‘‘‘You are tasked with analyzing social media posts for signs that may indicate clinical depression. You do not need to provide help or advice. Your role is to analyze and classify based on keywords, symptoms, or emotional expressions, without offering recommendations or assistance.  Treat news articles related to depression, research findings discussing depression or anxiety, and personal accounts displaying depression-related symptoms as indicative of clinical depression.  Follow these steps:  1. Identify if the post is a news article, research finding, or personal account.  2. Analyze the post for any keywords, symptoms, or emotional expressions that may be linked to clinical depression (e.g., hopelessness, fatigue, sadness, anxiety, or stress).  3. Evaluate the context:  - Posts that describe personal experiences of depression or symptoms (e.g., "I feel hopeless," "I'm struggling with sadness") should be classified as "YES."  - News articles or research findings directly discussing clinical depression, depression-related symptoms, or mental health issues (e.g., "depression," "anxiety") should be classified as "YES."  - Posts that mention depression in passing or discuss depression prevention without personal or symptomatic context (e.g., "Exercise helps prevent depression") should be classified as "NO."  4. Decide if the post is related to clinical depression by answering with a "YES" or "NO":  - Choose "YES" if the post directly mentions clinical depression, depressive symptoms, anxiety, or emotional distress related to mental health (e.g., hopelessness, fatigue, sadness, anxiety).  - Choose "NO" if the post discusses depression in a general or abstract way (e.g., news about preventing depression) without describing any depressive symptoms.  - Choose "NO" if the post is empty, too short to comprehend, or incomprehensible.  5. Provide the keywords or symptoms detected, if any.  6. Explain your decision briefly in one or two sentences, providing specific evidence for your classification.  7. For posts that are ambiguous or vague, lean toward a "NO" decision unless strong evidence suggests clinical depression.  **Important**: Do not provide any advice, support, or suggestions (e.g., hotlines). Focus strictly on analyzing the post and classifying it.  **Output Format**:  Please provide your response in the following structured format:  `["YES/NO", "<keywords/symptoms detected>", "<brief explanation of reasoning>"]`’’’ |

### BERTopic Parameters

Table S4. Details of parameters used in BERTopic. Note that parameters not specified were kept at their default values. Random seeds were fixed at 42 for reproducibility.

| **Step** | **Parameter** | **Values** |
| --- | --- | --- |
| Vectorization | stop_words | "english" |
| c-TF-IDF | reduce_frequent_words | **True** |
| Topic Representation | reduce_frequent_words | **True** |
|  | MaximalMarginalRelevance | diversity**=**0.9 |
|  | nr_topics | 25 |
