# Supplementary material for: What Does Text Mining of Reddit Forums Reveal About Factors Surrounding Mental Health in Singapore?
Source: J Med Internet Res. 2025 Oct 31;27:e72959. doi: 10.2196/72959 (PMC12578359; doi:10.2196/72959)

## Multimedia Appendix 2.

## Supplementary Results

### Linguistic Inquiry and Word Count (LIWC)

Figure S1. LIWC of Motives. The vertical dashed line represents the average score across reddit forums from the Test Kitchen Corpus.


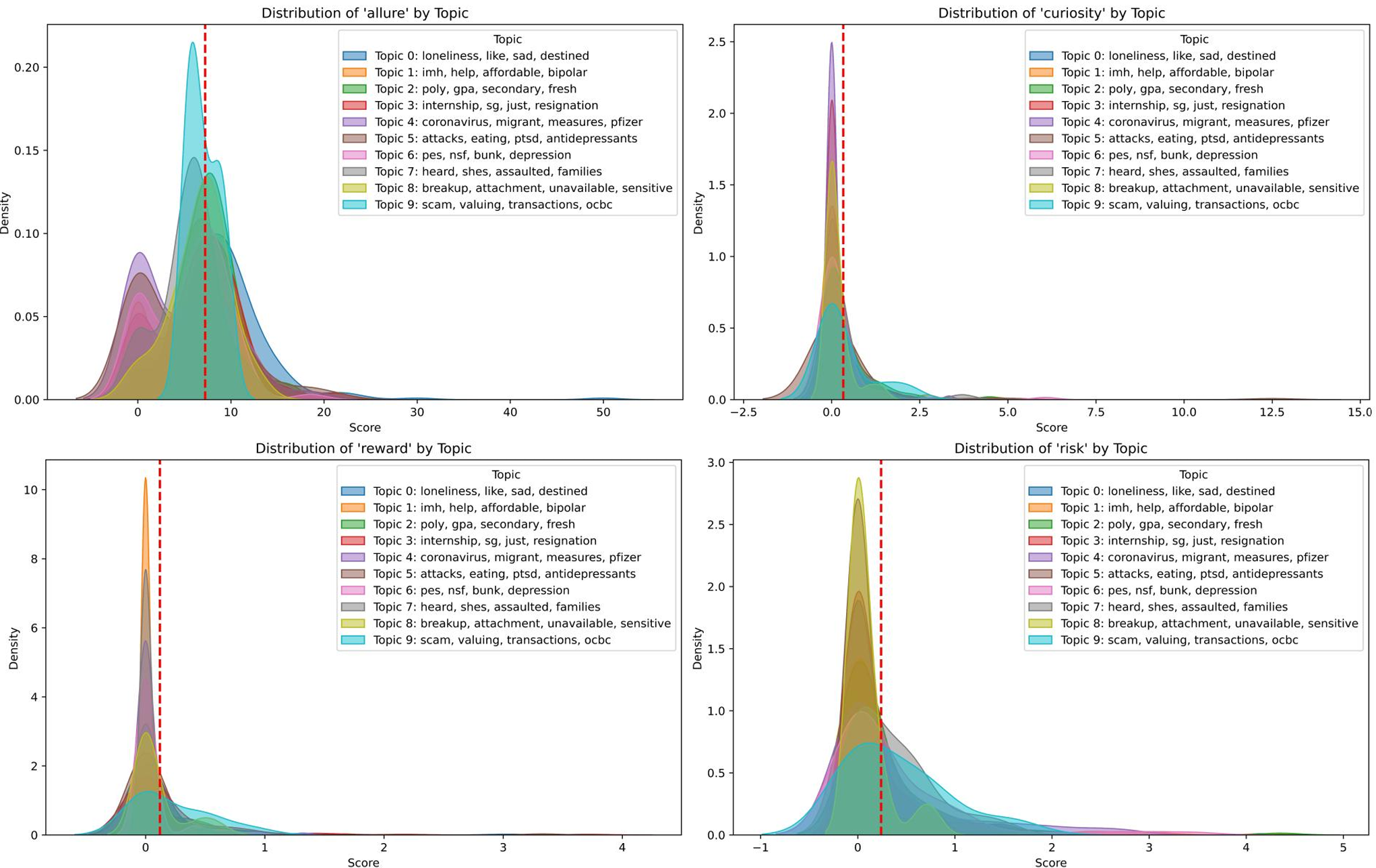


Figure S2. LIWC of States. The vertical dashed line represents the average score across reddit forums from the Test Kitchen Corpus.


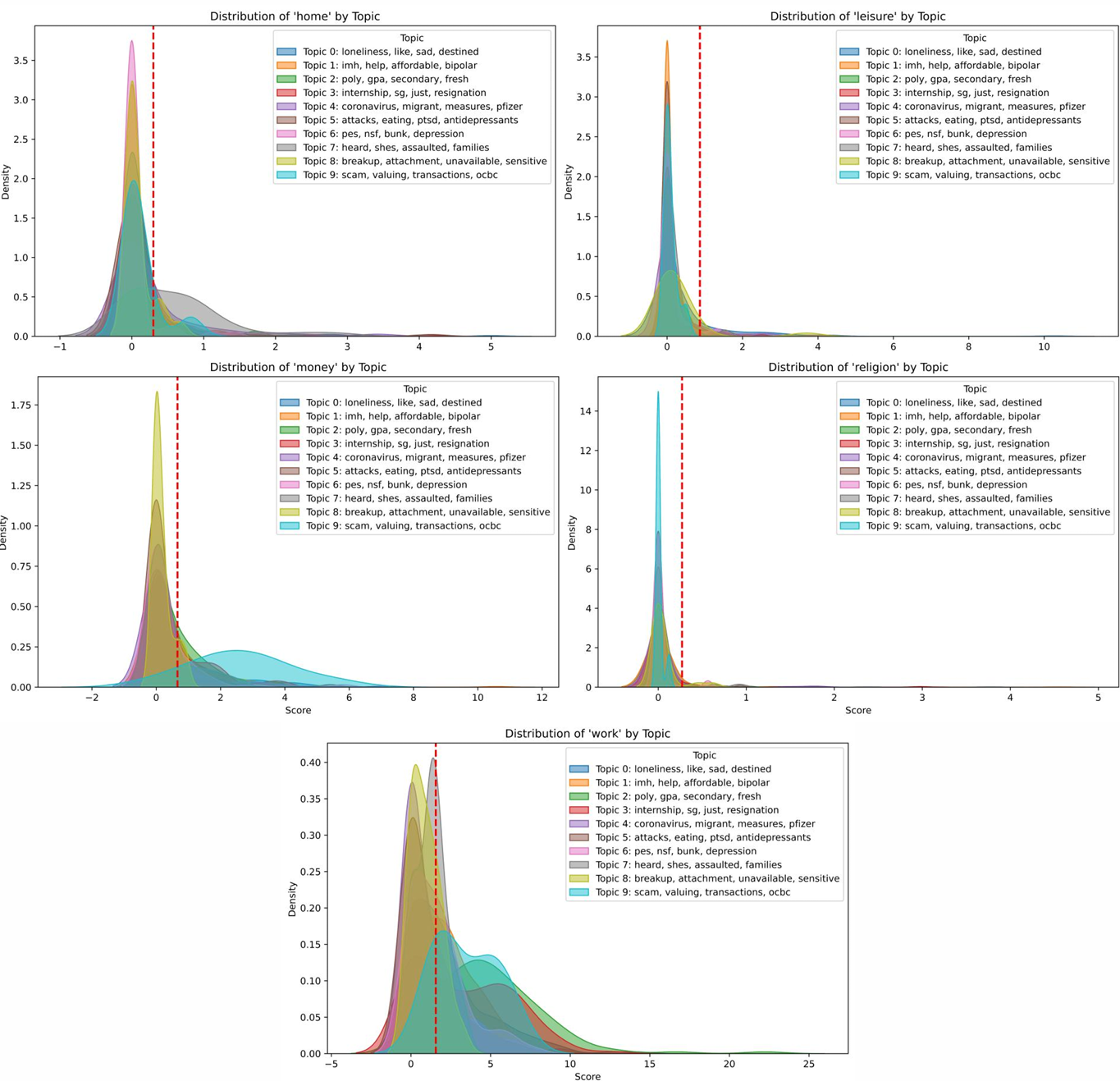


Figure S3. LIWC of Motives. The vertical dashed line represents the average score across reddit forums from the Test Kitchen Corpus.
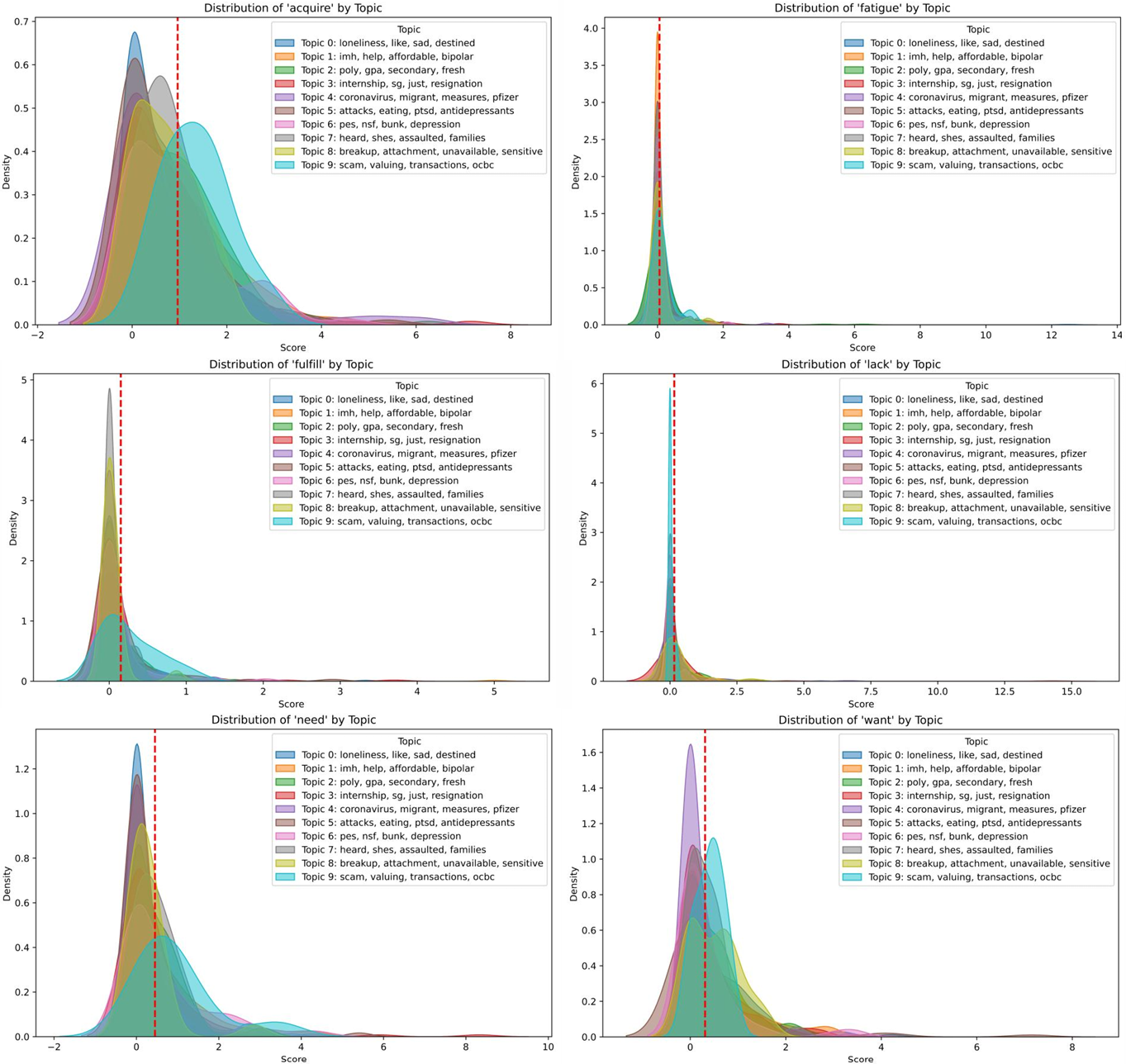

Supplement: Multimedia Appendix 2 [file jmir-v27-e72959-s002.docx]
